# Silicone Resin Coating of Micro-Sized Ferrite Particles Using Supercritical Carbon Dioxide

**DOI:** 10.3390/polym12092012

**Published:** 2020-09-03

**Authors:** Idzumi Okajima, Tatsuya Kanie, Takeshi Sako

**Affiliations:** 1 Department of Applied Chemistry and Biochemical Engineering, Faculty of Engineering, Shizuoka University, Hamamatsu 432-8561, Japan; okajima.izumi@shizuoka.ac.jp (I.O.); tatsuya_kanie@mail.toagosei.co.jp (T.K.); 2 Graduate School of Science and Technology, Shizuoka University, Hamamatsu 432-8561, Japan

**Keywords:** supercritical carbon dioxide, solubility, silicone resin, polymer coating, micro-sized particle

## Abstract

An environmentally friendly and efficient polymer coating method for micro-sized particles was developed using supercritical CO_2_. Because this method used supercritical CO_2_ as the solvent to dissolve the coating material, we avoided environmental pollution from organic solvents, saved the energy required to evaporate/remove organic solvents, realized a uniform coating film on the fine particles, and prevented agglomeration of the coating particles. The solubilities of the five silicone resins used as coating materials were measured using the flow method, and the data were well correlated by Chrastil’s equation with an average deviation of 5.7%. Resins comprising numerous methyl-group side chains exhibited high solubilities and were suitable coating materials. A new semi-flow-type coating method using supercritical CO_2_ was also developed, which deposited a film with a uniform thickness of 0.2–1.3 μm on whole fine particles. Notably, in this method, the film thickness was easily controlled. A simple and rapid technique was developed for measuring the coating thickness using X-ray fluorescence analysis. The model for calculating the coating film thickness was based on the material balance of the coating material. This model satisfactorily predicted the thickness with an average error of 0.085 μm by measuring the solubility of the coating material in supercritical CO_2_, integrated flow volume of supercritical CO_2_, particle diameter, density and charged weight of the fine particle, and coating material density.

## 1. Introduction

Polymer coating is important for surface functionalization and fabricating composite coatings of micro- and nano-sized particles. This technology is used in many fields, such as medicine, food, cosmetics, smart materials, and electronics; the most notable application is in drug delivery [1,2], in which the dissolution time of a drug in the human body is controlled, and the internal absorption or decomposition of the drug is suppressed by encapsulating it in a harmless polymer. Furthermore, this technology has been used to encapsulate food to avoid the oxidation of flavor compounds and lipids [3], and in surface modification to stabilize nano-sized particles with large surface energies in the field of electronics [4].

Supercritical CO_2_ has been studied in several fields because it is an environmentally friendly solvent that acts as a promising alternative to toxic and flammable organic solvents. Moreover, it has moderate critical constants (*T*_c_ = 304.3 K, *P*_c_ = 7.38 MPa), large solvating power for organic materials, low viscosity, high diffusivity, and near-zero surface tension [5]. These properties are suitable for the polymer coating of micro- to nano-sized particles because supercritical CO_2_ can dissolve the coating material easily and rapidly, and deliver it to the immediate vicinity of particle surfaces. Thus, the use of supercritical CO_2_ enables the production of polymeric materials with fillers, in which micro- or nano-sized fillers are dispersed in the polymer [6]. Further, it facilitates the fabrication of polymer-coated particles, in which organic solvent-free small particles coated by the polymer exist without agglomeration [7,8,9].

Polymer coating technologies have long employed wet coating methods. These methods use numerous solvents to dissolve coating polymers or monomers. Moreover, coating materials are usually hydrophobic and require organic solvents, which can pollute the environment and require significant energy for their evaporation and removal from the coating materials. The most commonly used wet coating method is the spray drying method [10], wherein the organic solution that dissolves the coating materials is sprayed onto fluidized particles that are then dried and coated. The advantages of this method are its simplicity and ease of operation, while its main disadvantage is that it is difficult to produce a thin and uniform coating film on the particles. The in-situ polymerization method is sometimes used for polymer coating [11]. In this method, small particles are dispersed in the organic solution, in which monomers of the coating polymer are dissolved; subsequently, the monomers are polymerized on the surfaces of the particles. The coated particles are then filtered and dried. This is also a simple and easy method; however, controlling the thickness of the coating film is difficult, and the coating particles often agglomerate during drying because of the surface tension of the solvent.

Silicone resins are non-toxic and possess excellent insulating, heat, and weather-resistant properties. Such resins are used in cosmetics and electrical wiring as coating and insulating materials, respectively. The spray drying method is often used industrially for silicone resin coating; however, problems, such as the agglomeration and non-uniformity of the film thickness of the coated materials, exist. Silicone resins are readily soluble in supercritical CO_2_. Therefore, the utilization of silicone resin coating using supercritical CO_2_ could help overcome the aforementioned problems.

For over 20 years, much attention has been paid to polymer coating technology using supercritical CO_2_. Typical applications of this method include encapsulation of drugs, bioactive materials, and cosmetic ingredients [12,13,14,15]. When encapsulating useful ingredients to protect against various factors, including oxygen, pH, heat, and light, organic solvents and heat treatment should be avoided to realize safety and high-performance. Furthermore, the development of uniform and thin coating techniques is required to control the release rate and position in the human body. Thus, an advanced coating technique using supercritical CO_2_ is necessary.

Another application is the synthesis of polymer-inorganic filler nanocomposites. In recent times, excellent properties and promising applications of these materials have been reported [16,17,18,19]. On mixing inorganic fillers with a polymer in supercritical CO_2_, the viscosity of the melting polymer is significantly decreased by the supercritical CO_2_, and the fillers disperse in the polymer effectively. This reduction in polymer viscosity and glass transition temperature in supercritical CO_2_ is an important phenomenon for polymer processing, including in our research.

The purpose of this study was the establishment of a coating technology using supercritical CO_2_ to produce a uniform coating film on micro-sized particles and to control and predict the film thickness. For this purpose, the following factors were examined: (1) Measuring the solubility of the coating polymer in supercritical CO_2_, in which the solubilities of five types of silicone resins were measured by the flow method and correlated using Chrastil’s equation. (2) The development of a semi-flow-type coating method, in which the applicability of a new coating method was examined to quickly and uniformly produce polymer coatings. (3) The development of a simple and quick method for measuring the thickness of the coating film; the usefulness of an X-ray fluorescence-based analysis was verified. (4) Establishing a model for calculating the thickness of the coating film. The model was based on the material balance of the coating polymer, and its applicability was investigated using the experimental data.

## 2. Materials and Methods

### 2.1. Materials

**Figure 1:**
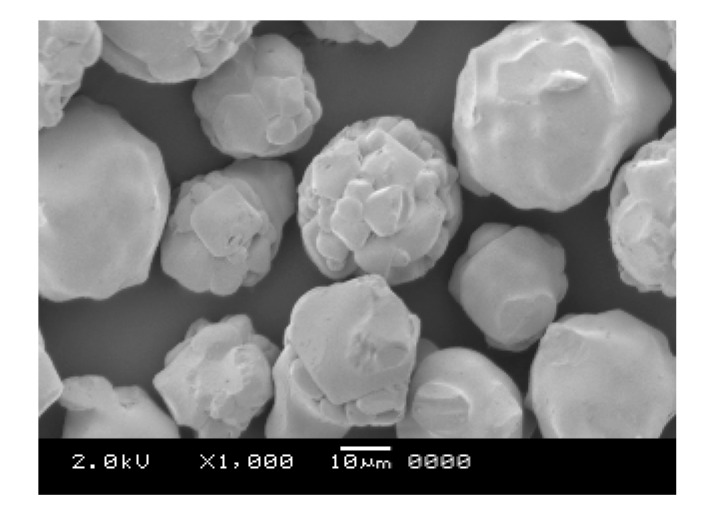
Scanning electron micrograph of the micro-sized ferrite particles (average diameter = 35 μm, true density = 4.89 g/cm^3^).

Micro-sized ferrite particles were used as the core material for coating in supercritical CO_2_. The particles were spherical and possessed irregularities, with an average particle size of 35 μm and a true density of 4.89 g/cm^3^. Figure 1 shows a scanning electron micrograph of the micro-sized ferrite particles. Ferrite fine particles were used as the core material for two main reasons. Firstly, the material is inexpensive, easily available, and widely used industrially as a magnetic material. Secondly, it is easy to observe the interface between the core material and silicone resin coating material when observing the cross-section of a coated ferrite particle by scanning electron microscopy (JSM-5600, JEOL Ltd., Tokyo, Japan). The micro-sized ferrite particles used in this work were provided by Ricoh Co., Ltd. (Tokyo, Japan).

Silicone resins were used as coating materials for the ferrite fine particles, and their solubilities in supercritical CO_2_ were measured. Such resins comprise siloxane bonds in the main chain and alkyl or phenyl groups in the side chain. The physical properties and structures of the five silicone resins used in this study are presented in Table 1 and Figure S1, respectively. The side chains of the five silicone resins comprise methyl and/or phenyl groups. Notably, since industrial silicone resins were employed in this study, their physical properties, such as molecular weight and glass transition point, differed slightly, depending on the product lot.

Carbon dioxide (99.5% purity; Air Liquide, Tokyo, Japan) was used as the solvent for the coating agent, while chloroform (special grade reagent, 99.5% purity; Wako Pure Chemicals Industries, Ltd., Osaka, Japan) was used for trapping the silicone resin, analysis, and equipment cleaning. Acetone (first-class reagent, 99% purity; Wako Pure Chemicals Industries, Ltd. Osaka, Japan) was used for equipment washing.

### 2.2. Solubility Measurements of the Coating Materials in Supercritical CO_2_

**Figure 2:**
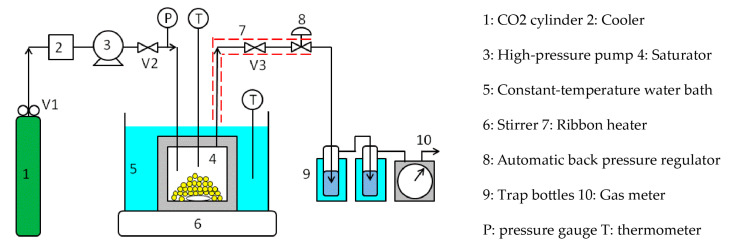
Flow-type apparatus used for the silicone resin solubility measurements.

Figure 2 shows a schematic diagram of the flow-type apparatus used for measuring the solubilities of the coating materials. The CO_2_ discharged from the cylinder (1) was cooled to 5 °C by the cooler (2), liquefied, and then introduced into the saturator (4) by the high-pressure pump (3). The saturator was an SUS316 cylindrical vessel with an inner diameter of 50 mm, depth of 65 mm, and inner volume of 125 cm^3^, and the maximum working pressure was 30 MPa. The coating material was placed in the saturator, which was immersed in a constant-temperature water bath (5) to control its temperature to an accuracy of ±0.1 °C. A stirrer (6) was installed to agitate the interior of the saturator. The CO_2_ in the saturator was heated above the critical temperature to achieve a supercritical state. The pressure was controlled to an accuracy of ±0.1 MPa by an automatic back pressure regulator (8) installed downstream of the saturator. The supercritical CO_2_ in the saturator dissolved the coating material to saturation solubility. The time required to reach dissolution equilibrium was reduced by stirring the interior of the saturator. After leaving the saturator, the supercritical CO_2_ containing the coating material was depressurized to atmospheric pressure by the automatic back pressure regulator, and the coating material was deposited and recovered in chloroform in the trap bottles (9). The weight of the coating material in chloroform was determined by Ultraviolet-visible spectrophotometry or High-Performance Liquid Chromatography, while the integrated flow volume of the CO_2_ at atmospheric pressure was measured by a gas meter (10). The piping from the constant-temperature water bath to the automatic back pressure regulator and the automatic back pressure regulator were maintained at approximately the same temperature as that of the constant-temperature water bath using a ribbon heater (7), so as not to precipitate the coating material from the supercritical CO_2_.

Briefly, the procedure for measuring the solubility of the silicone resin is as follows: approximately 5 g of the silicone resin was charged into the saturator, and high-pressure CO_2_ from the CO_2_ cylinder was introduced into the saturator. The saturator temperature and pressure were increased to the aforementioned values using the constant-temperature water bath and high-pressure pump. Subsequently, the saturator was closed by closing valves 2 and 3, after which it was stirred for 2–3 h so that the supercritical CO_2_ and silicone resin were in dissolution equilibrium. Valves 2 and 3 were then opened to allow the supercritical CO_2_, at the same temperature and pressure as that of the saturator, to flow through the saturator at a flow rate of approximately 0.5 mL/min for 3–5 h. The silicone resin dissolved in supercritical CO_2_ was then collected in chloroform in the trap bottles. Subsequently, the weight of the silicone resin in chloroform was determined by UV/Vis spectrophotometry (absorption of ~260 nm), while the total volume of CO_2_ passing through the saturator was measured with the gas meter. The solubility(s) of the solutes in supercritical CO_2_ is often expressed in mole fractions. However, since the silicone resin in this work had a molecular weight distribution, its solubility was expressed in [g/L] by the equation:

(1) $$s\;\left[g/L\right]=\;\frac{\mathrm{Weight}\;\mathrm{of}\;\mathrm{silicone}\;\mathrm{resin}\;\left[g\right]}{{\mathrm{Total}\;\mathrm{volume}\;\mathrm{of}\;\mathrm{supercritical}\;\mathrm{CO}}_{2}\;\mathrm{at}\;\mathrm{the}\;\mathrm{measured}\;\mathrm{temperature}\;\mathrm{and}\;\mathrm{pressure}\;\mathrm{conditions}\;\left[L\right]},$$

where the total volume of supercritical CO_2_ at the measured temperature and pressure was calculated from the total volume of CO_2_ measured at room temperature and atmospheric pressure, using the equation of state that relates the pressure, volume, and temperature of pure gases [20]. The flow method has the advantage of determining the solubility of any sample accurately; however, it requires a long measurement time and a specified amount of sample for highly accurate results.

### 2.3. Fine Particle Coating Using Supercritical CO_2_

**Figure 3:**
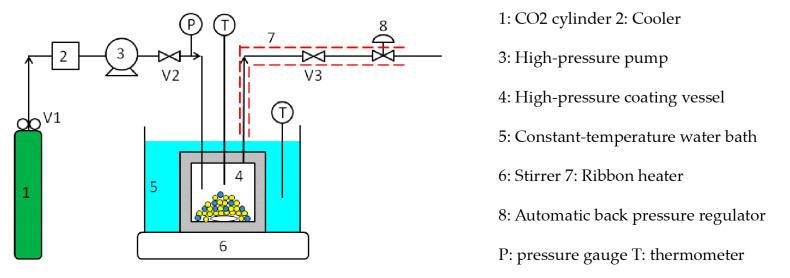
Batch-type supercritical coating apparatus.

Figure 3 shows a batch-type supercritical coating apparatus using supercritical CO_2_.

This is the apparatus in which the trap bottles and gas meter located downstream were removed from the solubility measuring apparatus in Figure 2. Briefly, the batch coating procedure is as follows: the micro-sized ferrite particles and silicone resin coating material were charged into a high-pressure coating vessel (4) and immersed in a constant-temperature water bath (5). Subsequently, valve 2 was opened and valve 3 was closed, and the supercritical CO_2_ was introduced into the coating vessel at a given pressure using the high-pressure pump (3). The high-pressure pump was then switched off, and the high-pressure coating vessel was closed by closing valve 2. The vessel was then stirred for 2–3 h until the coating material was completely dissolved. Subsequently, valve 3 was slightly opened and the coating vessel was depressurized at a constant rate of 0.28–2.08 MPa/min using the automatic back pressure regulator (8). After reducing to atmospheric pressure, the coating vessel was opened, and the coated ferrite fine particles were collected with a magnet.

**Figure 4:**
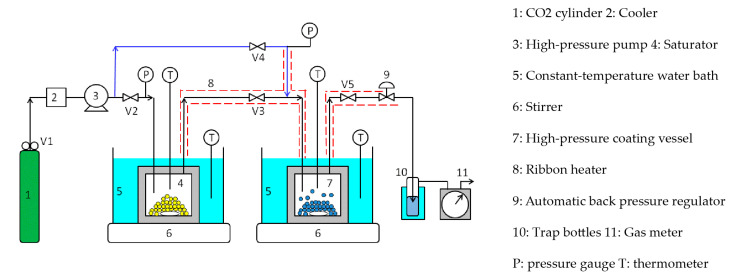
Semi-flow-type supercritical coating apparatus.

Figure 4 shows a semi-flow-type supercritical coating apparatus. Unlike the batch-type apparatus, this apparatus comprises a coating material saturator upstream of the coating vessel and piping (blue line) to introduce pure CO_2_ into the coating vessel. The semi-flow coating procedure is as follows: a large excess of silicone resin was charged in the saturator (4) and the ferrite fine particles were loaded in the coating vessel (7); both vessels were immersed in constant-temperature water baths (5) to maintain the same temperature. Ribbon heaters (8) were attached to the lines between the saturator and coating vessel and between the coating vessel and automatic back pressure regulator (9) and were heated to the same temperature as that of the saturator and coating vessel. Subsequently, valve 2 was opened while valve 3 was closed, and the supercritical CO_2_ was introduced into the saturator to a given pressure using the high-pressure pump (3). Subsequently, the high-pressure pump was switched off, and the saturator was closed by closing valve 2. The vessel was then stirred for 2–3 h until the coating material was dissolved to saturation solubility. Valve 4 was then opened while valve 5 was closed, and high-pressure CO_2_ was introduced into the coating vessel through the blue line to create supercritical CO_2_ at the same temperature and pressure as that of the saturator. The coating vessel was then closed by closing valve 4 and stirred subsequently. After the supercritical CO_2_ dissolved the coating material to saturation solubility, valves 2, 3, and 5 were opened. While maintaining the dissolution equilibrium between the supercritical CO_2_ and silicone resin in the saturator, high-pressure CO_2_ was supplied to the saturator at a low flow rate of 0.5 mL/min using the high-pressure pump, and the supercritical CO_2_ with silicone resin in the saturator was driven out to the coating vessel. After charging the supercritical CO_2_-dissolved silicone resin into the coating vessel for a certain period (4–30 min), valves 2, 3, and 5 were closed to seal the coating vessel. The coating vessel was then stirred for 15 min until the concentration of the silicone resin in the vessel became completely uniform. Subsequently, valve 5 was opened and the pressure in the coating vessel was reduced to atmospheric pressure at a constant rate of 0.42 MPa/min, using the automatic back pressure regulator to coat the silicone resin on the surface of the ferrite fine particles. At the end of the experiment, the ferrite fine particles in the coating vessel were collected with a magnet.

### 2.4. Measurement of the Coating Film Thickness

The thickness of the silicone resin film coated on the ferrite fine particles was measured by two methods: X-ray fluorescence analysis and cross-sectional scanning electron microscopy. The former measures the X-ray fluorescence intensity emitted from the silicon present in the silicone resin and the iron in the ferrite fine particles using a scanning X-ray fluorescence spectrometer (ZSX100e, Rigaku Co., Tokyo, Japan), with film thickness determined from the silicon-to-iron X-ray fluorescence intensity ratio (Si/Fe). In the latter, film thickness was measured by observing the cross sections of ferrite fine particles before and after coating by field-emission scanning electron microscopy (JSM-7001FTTLS, JEOL Ltd., Tokyo, Japan).

## 3. Results and Discussion

### 3.1. Solubility of the Silicone Resins in Supercritical CO_2_

#### 3.1.1. Solubility Measurements

The solubilities of the five silicone resins (Table 1) were measured in supercritical CO_2_ using the flow method. Before measuring the solubilities, the relationship between the supercritical CO_2_ flow rate and solubility was examined to determine the appropriate supercritical CO_2_ flow rate. Figure S2 shows the experimental results for resin S1. The solubility of this resin did not depend on the flow rate of supercritical CO_2_ in this flow rate range, because dissolution equilibrium was achieved in the saturator across the entire flow rate range. Indeed, the results revealed that supercritical CO_2_ flow rates ≤0.9 mL/min were suitable for measuring the solubilities of the silicone resins. To maintain dissolution equilibrium, a flow rate of 0.5 mL/min was selected in this study.

**Figure 5:**
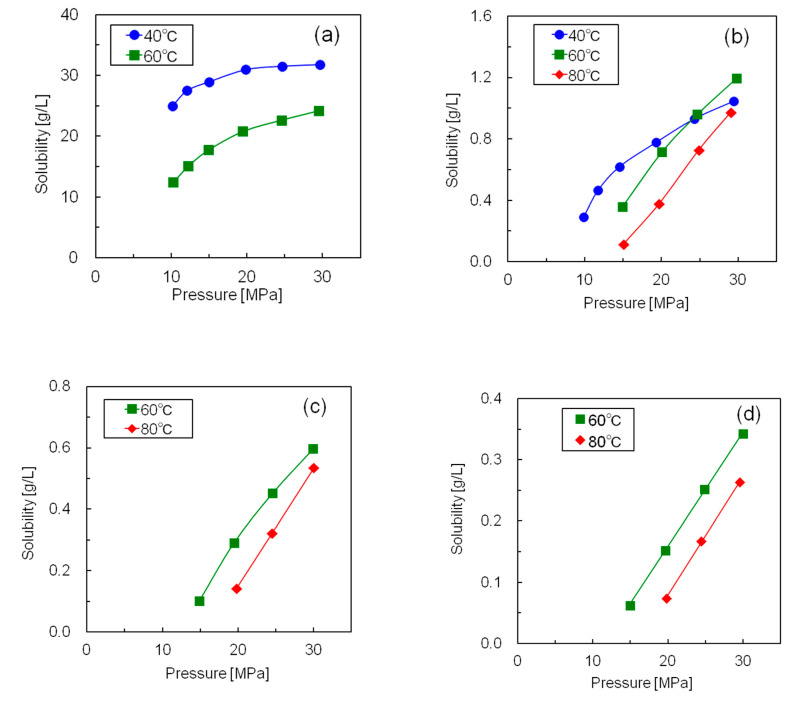

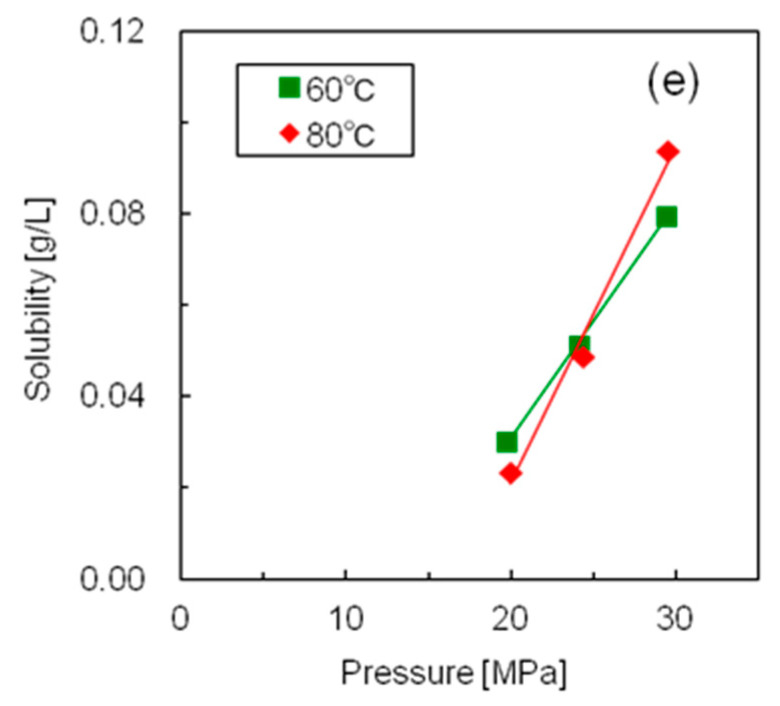
Solubilities of the five silicone resins at different temperatures and pressures in supercritical CO_2_ (**a**) Resin S1; (**b**) Resin S2; (**c**) Resin S3; (**d**) Resin S4; (**e**) Resin S5.

Figure 5 shows the measured solubilities of the five silicone resins in supercritical CO_2,_ and Table S1 presents the numerical solubility data of all the resins. Since all the silicone resins displayed molecular weight distributions, the solubilities are expressed in mass units [g/L] rather than molar units (mol/L). Although the solubilities of all silicone resins increased with increasing pressure, the temperature dependence of the solubility was complex. The isothermal solubility curves of resin S2 at 40 and 60 °C, and those of resin S5 at 60 and 80 °C intersected at ~24 MPa. The solubility increased with increasing temperature above the intersection pressure and decreased with increasing temperature below this pressure. On the contrary, the solubilities of resins S1, S3, and S4 decreased with increasing temperature across the whole pressure range. This could be explained by the difference between the temperature dependence of the CO_2_ density and that of the vapor pressure of the solute [21]. The solubility of the solute increased with increasing solute vapor pressure and CO_2_ density. Moreover, the vapor pressure of the silicone resin increased with increasing temperature; however, the increment was minimal because silicone resin has a large molecular weight and two-dimensional structure. On the contrary, the density of supercritical CO_2_ was greatly reduced by the temperature rise in the low-pressure region. As a result, CO_2_ exhibited a large negative density effect, and the solubility was reduced by the temperature rise in the low-pressure region. Conversely, the decrease in CO_2_ density was minimal in the high-pressure region with increasing temperature. As a result, the silicone resin showed a large positive effect on vapor pressure, with solubility increasing with rising temperature in the high-pressure region. Thus, we concluded that low temperature was advantageous for realizing high silicone resin solubility under the same pressure.

**Figure 6:**
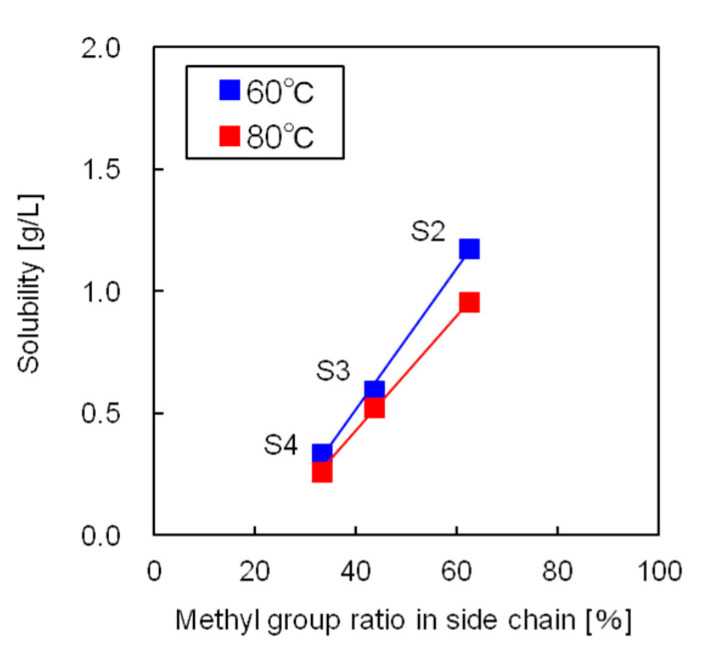
Influence of the side chain methyl group ratio on silicone resin solubility.

Figure 6 shows the influence of the side chain methyl-to-phenyl group ratio on the solubilities of the silicone resins. The free volume of the silicone resin increased as the ratio of methyl groups, which are significantly less bulky than the phenyl groups, increased. As a result, the CO_2_ molecules in the supercritical state could move and solvate the silicone resin more easily. Thus, the silicone resin with the highest number of methyl groups showed the highest solubility in supercritical CO_2_.

#### 3.1.2. Solubility Correlation

The solubility data for the silicone resins in supercritical CO_2_ were correlated using Chrastil’s equation [22]:

(2) $$\ln S=\alpha \ln\rho_{1}+\frac{\beta }{T}+\gamma ,$$

where S is the solubility of the silicone resin, $\rho_{1}$ is the density [g/L] of supercritical CO_2_ under the experimental temperature and pressure conditions, *T* is the absolute temperature [K],  and α, β, and γ are constants. This equation was derived based on the assumption that the solute molecules and supercritical solvent molecules associate to form solvated complexes. The constants α, β, and γ are temperature and pressure independent and were determined by fitting the solubility data to Equation (2) by the least-squares method. The objective function *F* used for the least-squares method is given by:

(3) $$F=\ln S_{\exp}-\ln S_{\mathrm{cal}}=\ln S_{\exp}-\left({\alpha \ln\rho }_{1}+\frac{\beta }{T}+\gamma \right),$$

where *S*_exp_ is the measured solubility, and *S*_cal_ is the calculated solubility. The constants α, β, and γ were determined so that the sum of the squares of the objective function *F* was minimized.

**Figure 7:**
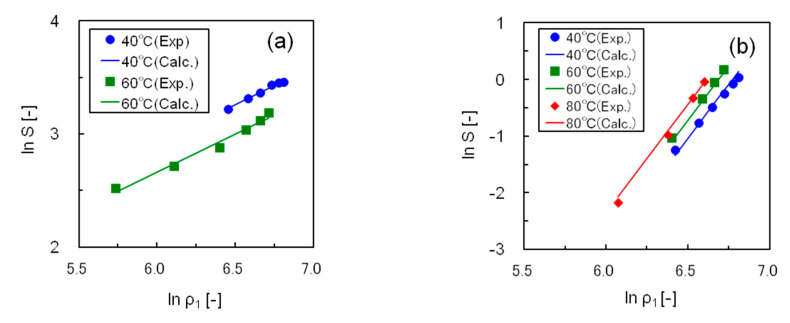
Correlating the solubility data for silicone resins using Chrastil’s equation (**a**) Resin S1; (**b**) Resin S2.

Table 2 shows the optimum values of α, β, and γ in Equation (2) and the correlation deviations for the five silicone resins. The solubility data of the five resins were correlated accurately, with an average deviation within 6%. Figure 7 compares the experimental and calculated solubilities of resins S1 and S2, in which the straight lines show the correlated results. In this figure, the isothermal plots of each resin at 40–80 °C are linear, having the same slope, and a suitable correlation was obtained using Equation (2).

### 3.2. Silicone Coating of the Micro-Sized Ferrite Particles

Of the five studied resins, S1 displayed a significantly high solubility in supercritical CO_2_ compared to the other silicone resins, together with low environmental load, low cost, and ease of availability. Thus, S1 was selected as the coating material for the subsequent silicone coating of the ferrite fine particles using supercritical CO_2_.

#### 3.2.1. Batch Coating Method

Batch coating of the micro-sized ferrite particles with silicone resin S1 was carried out using the apparatus shown in Figure 3. After coating, the cross sections of the coated ferrite fine particles were observed to determine the coating film thickness, and the dependence of film thickness on the pressure reduction rate was examined. Furthermore, a simple method to determine the coating film thickness was developed.

**Figure 8:**
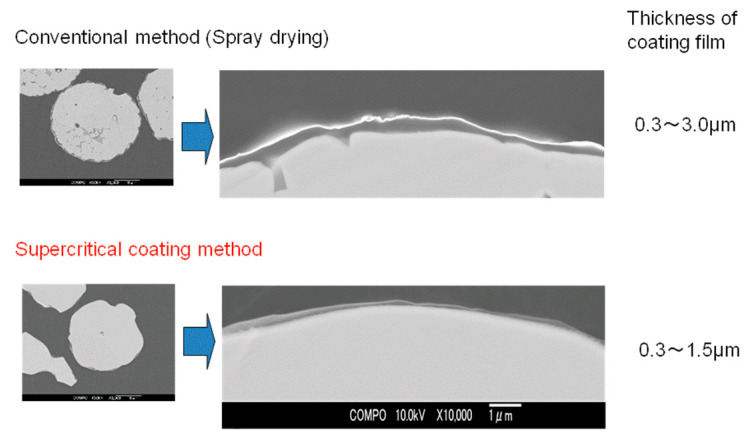
Scanning electron micrographs of the cross sections of ferrite fine particles coated with silicone resin S1 using conventional and supercritical coating methods at 40 °C and 25 MPa.

Figure 8 shows scanning electron micrographs of the cross sections of ferrite fine particles coated with silicone resin S1. The upper image shows ferrite fine particles coated by the conventional spray drying method, while the lower image shows particles coated by the supercritical CO_2_ method developed in this study. Images of the entire particle, and the vicinity of the interface between the ferrite particle and silicone resin have also been shown. The ferrite fine particles coated with supercritical CO_2_ had thinner films than those coated by the spray drying method; however, the former method coated the entirety of the fine particles smoothly and uniformly. This was because approximately the same amount of coating material per surface area was deposited on every fine particle surface under reduced pressure, thus forming a uniform coating film.

The relationship between the coating film thickness and supercritical CO_2_ decompression rate was examined, and it was observed that the thickness of the coating film did not depend on the supercritical CO_2_ decompression rate. This observation is explained as follows: As the decompression rate changed, the deposition rate of the coating material also changed, while the deposition amount did not. Furthermore, we estimated that the ratio of deposition on the fine particles to the nucleation of the coating material did not change on altering the pressure reduction rate.

**Figure 9:**
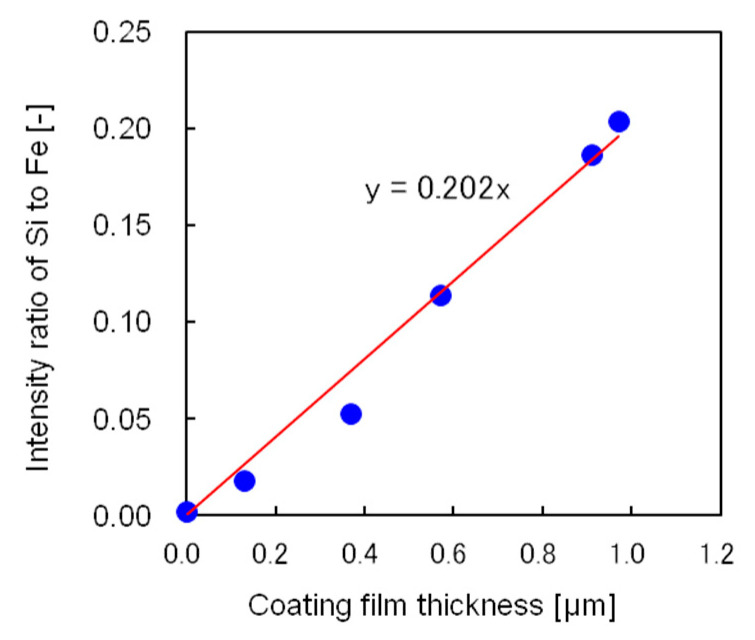
Relationship between the silicon-to-iron X-ray fluorescence intensity ratio and coating film thickness measured from the cross-sectional scanning electron micrograph.

A simple and accurate method to determine the film thickness was also developed. Figure 9 shows the relationship between the silicon-to-iron X-ray fluorescence intensity ratio and film thickness measured from cross-sectional scanning electron micrographs. The relationship is well represented by a straight line passing through the origin; hence, the coating film thickness could be easily determined by X-ray fluorescence analysis using this straight line.

#### 3.2.2. Semi-Flow Coating Method

**Figure 10:**
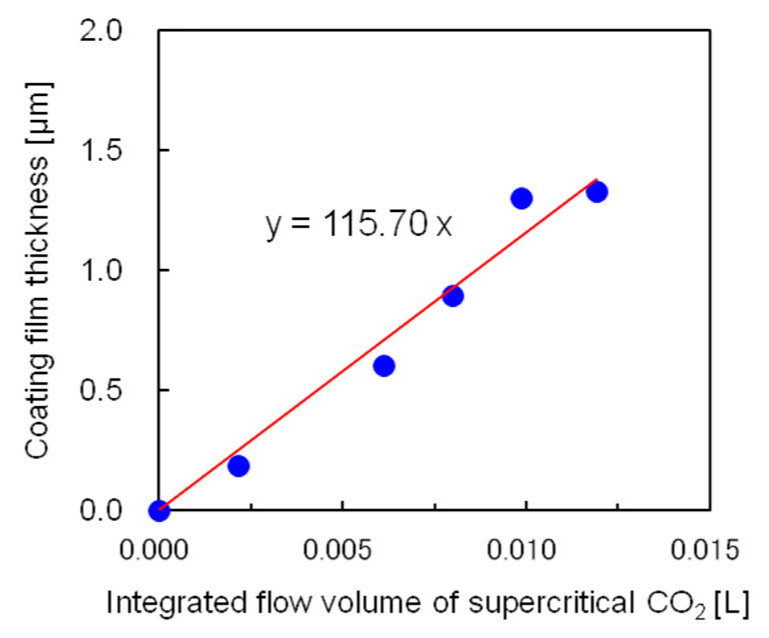
Dependence of the coating film thickness on the integrated flow volume of supercritical CO_2._ at 40 °C and 25 MPa using the semi-flow coating method.

Using the semi-flow coating method, the relationship between the thickness of the silicone coating film and integrated flow volume of the supercritical CO_2_ used to dissolve the saturated coating material was studied next. Figure 10 shows the experimental results, in which the x-axis represents the integrated flow volume of supercritical CO_2_ saturated by the coating material at 40 °C and 25 MPa. While calculating the integrated flow volume of the supercritical CO_2_, it was necessary to subtract 2 cm^3^ of supercritical CO_2_ in the tubing between the saturator and coating vessel because the supercritical CO_2,_ devoid of coating material present in the tubing, diffused to the coating vessel immediately after it started flowing. In the semi-flow coating method, the coating film thickness was proportional to the integrated flow volume of the supercritical CO_2_ and could be adjusted by controlling the integrated flow volume. As a result, a film thickness greater than that produced by the batch method could be obtained.

#### 3.2.3. Model for Calculating the Coating Film Thickness in the Semi-Flow Coating Method

A calculation model for the film thickness was devised for the semi-flow coating method. The following assumptions were considered to create the model: (1) The fine particles are spheres with the same diameter. (2) The film thickness is uniform over the entire fine particle. (3) The inner wall of the coating vessel is coated to the same thickness as that of a fine particle. (4) The outflow of the coating material from the coating vessel is proportional to the inflow of the coating material to the coating vessel.

The equations to calculate the coating film thickness were derived based on the material balance of the coating material in the coating vessel. The coating material flowing into the coating vessel is equal to the sum of the coating material flowing out of the coating vessel and that present on the fine particles and inner wall of the coating vessel:

(Inflow of coating material to coating vessel) = (outflow of coating material from coating vessel) + (coating material on fine particles) + (coating material on inner wall of coating vessel).(4)

The inflow of the coating material to the coating vessel is represented by the product of solubility *S* of the coating material and the time integral of the flow rate *F*:

(5) $$\left(\mathrm{Infow}\right)=S\int_{0}^{t}Fdt.$$

When the flow rate is constant with respect to flow time *t*, Equation (5) can be expressed by Equation (6):

(6) (inflow)=SFt.

Since the outflow of the coating material is proportional to the inflow of the coating material, this relationship can be expressed by the equation:

(7) (Outflow)=kSFt,

where k is the proportionality constant determined from the experimental data.

The weight of the resin coated on the fine particles was calculated by the equation:

(8) $$\left(\mathrm{Weight}\;\mathrm{of}\;\mathrm{resin}\;\mathrm{coated}\;\mathrm{on}\;\mathrm{particles}\right)=\left\{\frac{\pi }{6}{\left(d_{h}+2C\right)}^{3}-\frac{\pi }{6}d_{h}^{3}\right\}\times \frac{I_{h}}{\frac{\pi }{6}d_{h}^{3}\rho_{h}}\times \rho_{c}\;=\left(\frac{6C}{d_{h}}+\frac{12C^{2}}{d_{h}^{2}}+\frac{8C^{3}}{d_{h}^{3}}\right)\frac{\rho_{c}I_{h}}{\rho_{h}}\;,$$

where the first term represents the volume of the resin coated on a fine particle, and was calculated by subtracting the volume of a fine particle from the volume of a coated fine particle; *d*_h_ is the diameter of the fine particle; *C* is the coating film thickness. The second term represents the number of fine particles, in which $I_{h}$ and $\rho_{h}$ are the weight and density of the fine particles, respectively. Finally, the third term $\rho_{c}$ is the density of the coating material. In Equation (8), (*C*/*d*_h_)^2^ and (*C*/*d*_h_)^3^ were negligible because the film thickness *C* is much smaller than the particle diameter *d*_h_. Thus, Equation (8) can be written as:

(9) $$\left(\mathrm{Weight}\;\mathrm{of}\;\mathrm{resin}\;\mathrm{coated}\;\mathrm{on}\;\mathrm{particles}\right)=\frac{6C\rho_{c}I_{h}}{d_{h}\rho_{h}}.$$

Subsequently, the weight of the resin coated on the inner wall was calculated by Equation (10):

(10) $$\begin{array}{c} \left(\mathrm{Weight}\;\mathrm{of}\;\mathrm{resin}\;\mathrm{coated}\;\mathrm{on}\;\mathrm{wall}\;\mathrm{of}\;\mathrm{coating}\;\mathrm{vessel}\right)\\ \;=\left\{\frac{\pi }{4}d_{v}^{2}L-\frac{\pi }{4}{\left(d_{v}-2C\right)}^{2}\left(L-2C\right)\right\}\times \rho_{c}\\ =\left\{2C^{3}-\left(L+2d_{v}\right)C^{2}+\left(d_{v}L+\frac{1}{2}d_{v}^{2}\right)C\right\}\times \pi \rho_{c}\;, \end{array}$$

where the first term represents the volume of resin coated on the inner wall of the cylindrical coating vessel, calculated by subtracting the inner volume of the coating vessel after coating from that before coating; *d*_v_ is the inner diameter of the cylindrical coating vessel, and *L* is the height of the coating vessel. In Equation (10), the terms equal or greater than *C*^2^ are negligible because *C* is much smaller than *d*_v_ and *L*. Thus, Equation (10) can be written as:

(11) $$\left(\mathrm{Weight}\;\mathrm{of}\;\mathrm{resin}\;\mathrm{coated}\;\mathrm{on}\;\mathrm{wall}\;\mathrm{of}\;\mathrm{coating}\;\mathrm{vessel}\right)=\left(d_{v}L+\frac{1}{2}d_{v}^{2}\right)\pi C\rho_{c}.$$

Introducing Equations (6), (7), (9), and (11) into Equation (4) gives:

(12) $$SFt=kSFt+\frac{6{C\rho }_{c}I_{h}}{d_{h}\rho_{h}}+\left(d_{v}L+\frac{1}{2}d_{v}^{2}\right)\pi C\rho_{c}.$$

Modifying Equation (12) for the film thickness *C* gives Equation (13):

(13) $$C=\frac{\left(1-k\right)S}{\frac{6\rho_{c}I_{h}}{d_{h}\rho_{h}}+\left(d_{v}L+\frac{1}{2}d_{v}^{2}\right)\pi \rho_{c}}Ft.$$

Replacing the solubility of the coating material S with Chrastil’s Equation (2) gives:

(14) $$C=\frac{\left(1-k\right)\exp\left(\alpha \ln\rho_{1}+\frac{\beta }{T}+\gamma \right)}{\frac{6\rho_{c}I_{h}}{d_{h}\rho_{h}}+\left(d_{v}L+\frac{1}{2}d_{v}^{2}\right)\pi \rho_{c}}Ft.$$

Equation (13) reveals that the coating film thickness is proportional to the solubility of the coating material, and the flow rate and time of the supercritical CO_2_.

To calculate the coating film thickness using Equation (14), it was necessary to determine the proportional constant k in the term for the outflow amount. Figure S3 shows the relationship between the inflow and outflow amounts of the coating material. Notably, the fourth assumption described at the beginning of this section was reasonable because a good proportional relationship was observed between both flows. The proportionality constant k was determined to be 0.463 from the slope of the straight line.

**Figure 11:**
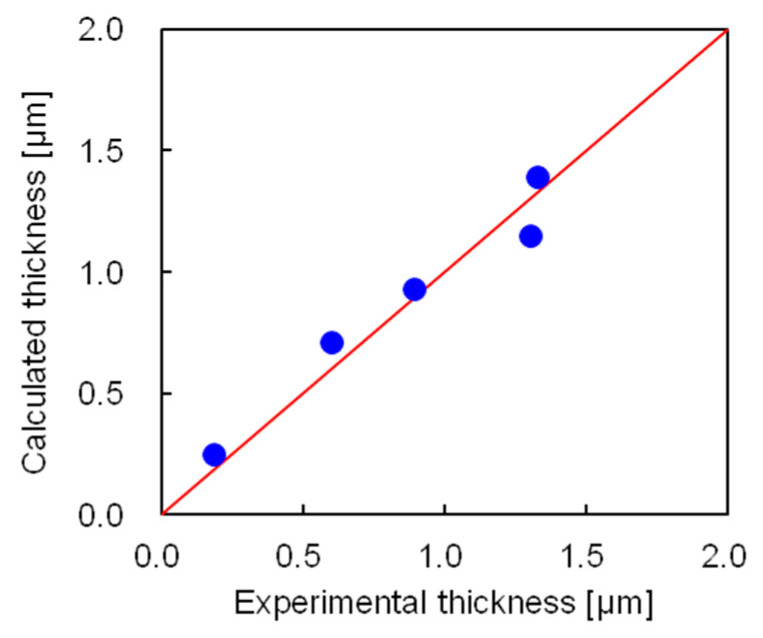
Comparing the experimental and calculated thicknesses of coating films.

The coating film thickness was calculated using Equation (14), where the numerical values are given in Table S2. Figure 11 compares the experimental and calculated thicknesses of the coating film. The calculated results agreed well with the experimental data, with an average error of 0.085 μm. Thus, we concluded that the model for determining the coating film thickness in the semi-flow coating method successfully calculated the experimental coating thickness. The ratio of the amounts of coating material in the outflow, on the ferrite fine particles, and on the inner wall was calculated using the developed model, which revealed that 46% of the coating material was driven out of the coating vessel, 46% was coated on the fine particles, and 8% was coated on the inner wall of the coating vessel.

## 4. Conclusions

A micro-sized particle coating technology using supercritical CO_2_ instead of conventional organic solvents was developed, and the solubilities of the coating materials in supercritical CO_2_ were measured to realize an environmentally friendly and high-performance coating technology. The solubilities of five silicone resins in supercritical CO_2_ were measured using the conventional flow method. The silicone resins showed retrograde phenomena below 24 MPa or when measured across the entire 10–30 MPa pressure range, in which higher solubilities were observed at lower rather than higher temperatures. This was explained qualitatively by the relationships between the density of supercritical CO_2_ and the silicone resin vapor pressure with temperature. Furthermore, the silicone resin solubility was significantly increased by replacing the phenyl groups, which occupy a large volume, with smaller methyl groups. The solubility data for the silicone resins were correlated using Chrastil’s equation, with a 5.7% average deviation between the measured and calculated results.

We also examined an easy way of determining the film thickness in the fine particle coating process using supercritical CO_2_. A new calibration curve for coating film thickness was obtained from the X-ray fluorescence analysis of the coated particle surface and the actual film thickness measured by field-emission scanning electron microscopy. This calibration curve enabled simple and quantitative evaluation of the film thickness using X-ray fluorescence data.

A new semi-flow coating method using supercritical CO_2_ was also developed. In this method, a large amount of coating material was efficiently dissolved in supercritical CO_2_ using a dissolving vessel capable of stirring, and the film thickness was controlled by the total flow volume of supercritical CO_2_ containing the coating material. A model for calculating the coating film thickness in the semi-flow coating method was established, in which the coating film thickness was expressed as a function of the coating material solubility, supercritical CO_2_ integrated flow volume, and charged amount of fine particles. The measured coating film thicknesses were compared with those calculated by the model, revealing good agreement with a mean deviation of 0.085 μm. This coating technology using supercritical CO_2_ could easily produce a uniform film with controlled-thickness and predict the film thickness accurately. Furthermore, as the semi-flow-type coating method is easy to scale up, it is expected to be applied in drug delivery and cosmetic processing.

## Figures and Tables

**Table 1:** Properties of the five silicone resins used in this study.

Sample No.	Side Chain	Methyl:Phenyl in Side Chain (%)	Weight-Average Molecular Weight (g/mol)	Density (g/cm^3^)	Glass Transition Temp. (°C)
S1	Methyl	100:0	2500	1.42	40
S2	Methyl-phenyl	62.5:37.5	3000	1.30	37.9
S3	Methyl-phenyl	43.5:56.5	3000	1.32	-
S4	Methyl-phenyl	33.3:66.7	3000	1.33	-
S5	Phenyl	0:100	2000	1.34	51.7

**Table 2:** Optimum values of Chrastil’s equation and average deviations.

Sample No.	α	β	γ	Ave. Deviation (%)
S1	0.6715	−1356	−5.437	1.9
S2	3.826	−1687	−20.53	6.0
S3	5.586	−2468	−30.61	2.7
S4	5.421	−2191	−30.93	1.9
S5	5.910	−4396	−29.07	6.2

## Supplementary Materials

The following supplementary materials are available online at https://www.mdpi.com/2073-4360/12/9/2012/s1: Figure S1: Structures of the five silicone resins used in this study, Figure S2: Relationship between the solubility of silicone resin S1 and the flow rate of supercritical CO_2_ at 40 °C and 25 MPa, Figure S3: Relationship between the inflow and outflow amounts of the coating material, Table S1: Solubilities of five silicone resins in supercritical CO_2_, Table S2: Numerical values used in the model to calculate the coating film thickness.
