# Supplementary material for: Silicone Resin Coating of Micro-Sized Ferrite Particles Using Supercritical Carbon Dioxide
Source: Polymers (Basel). 2020 Sep 3;12(9):2012. doi: 10.3390/polym12092012 (PMC7565151; doi:10.3390/polym12092012)
Supplement: Supplementary file 1 [file polymers-12-02012-s001.pdf]

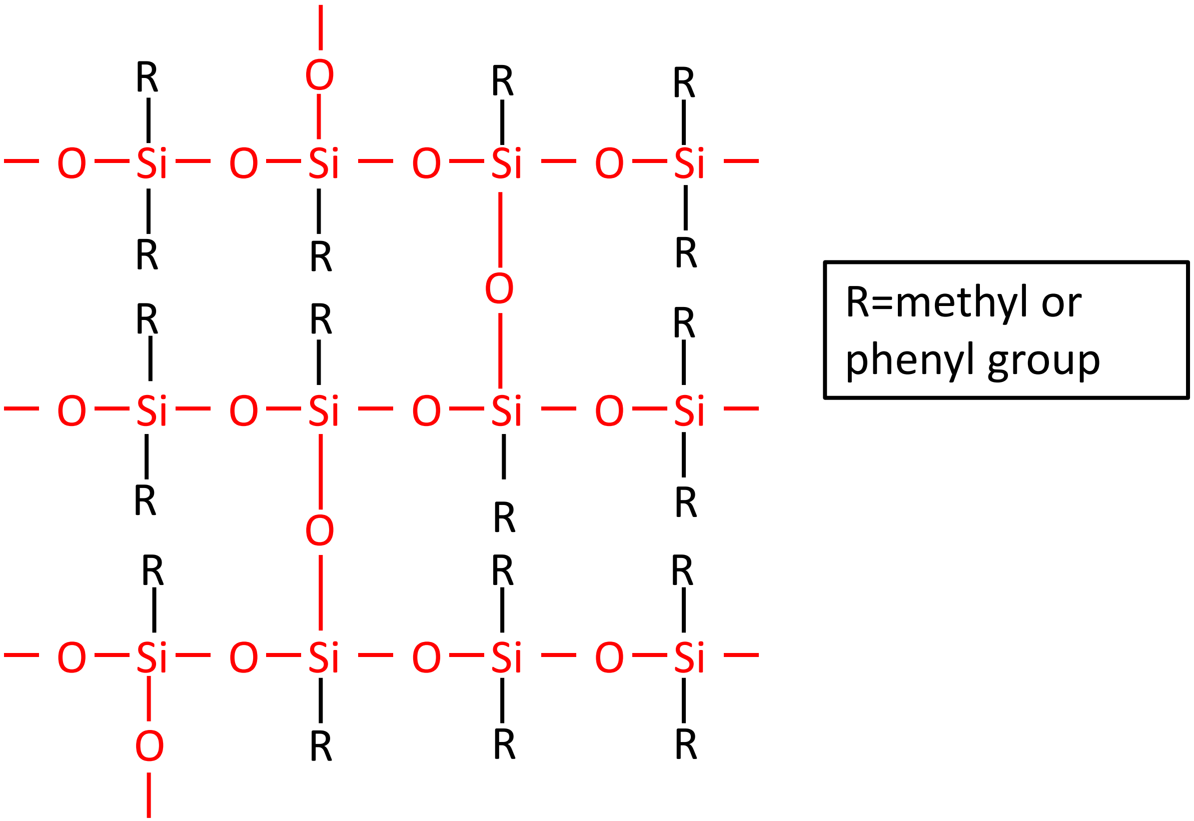

Figure S1. Structures of the five silicone resins used in this study

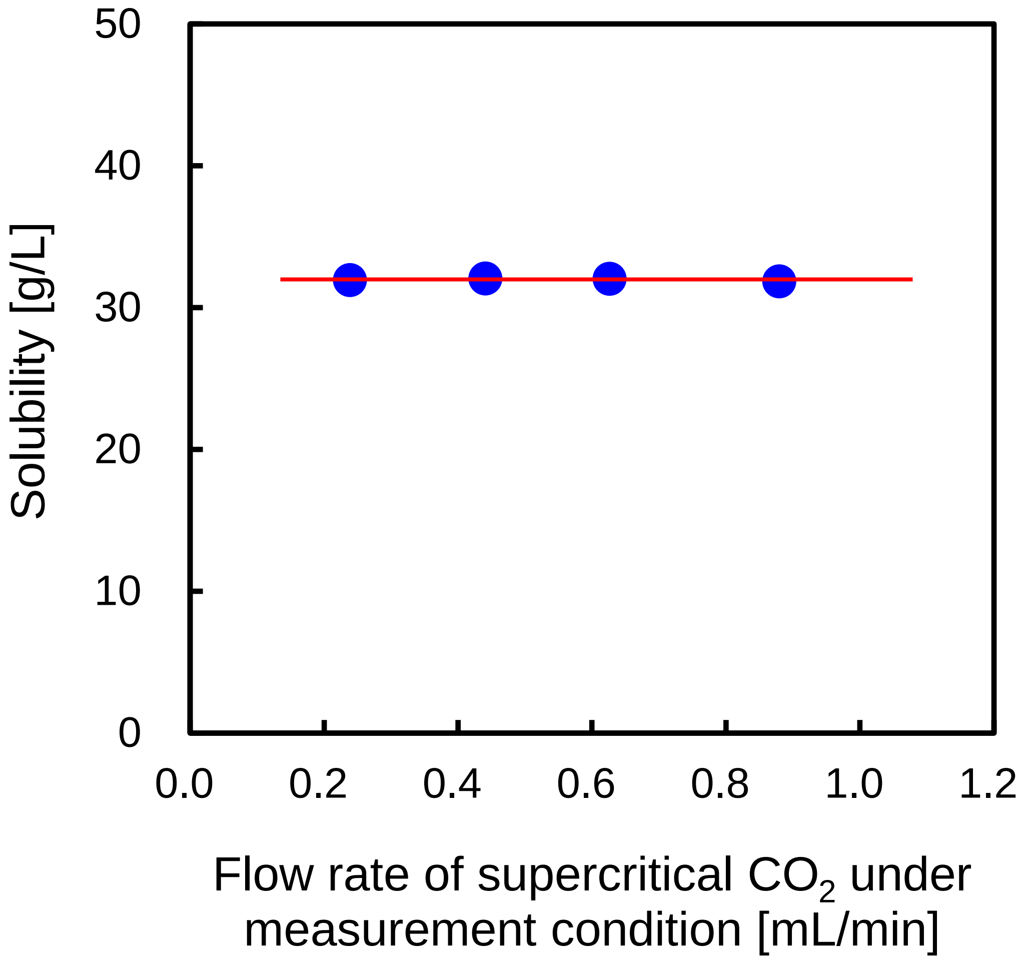

Figure S2. Relationship between the solubility of silicone resin S1 and the flow rate of supercritical CO_2 at 40℃ and 25MPa

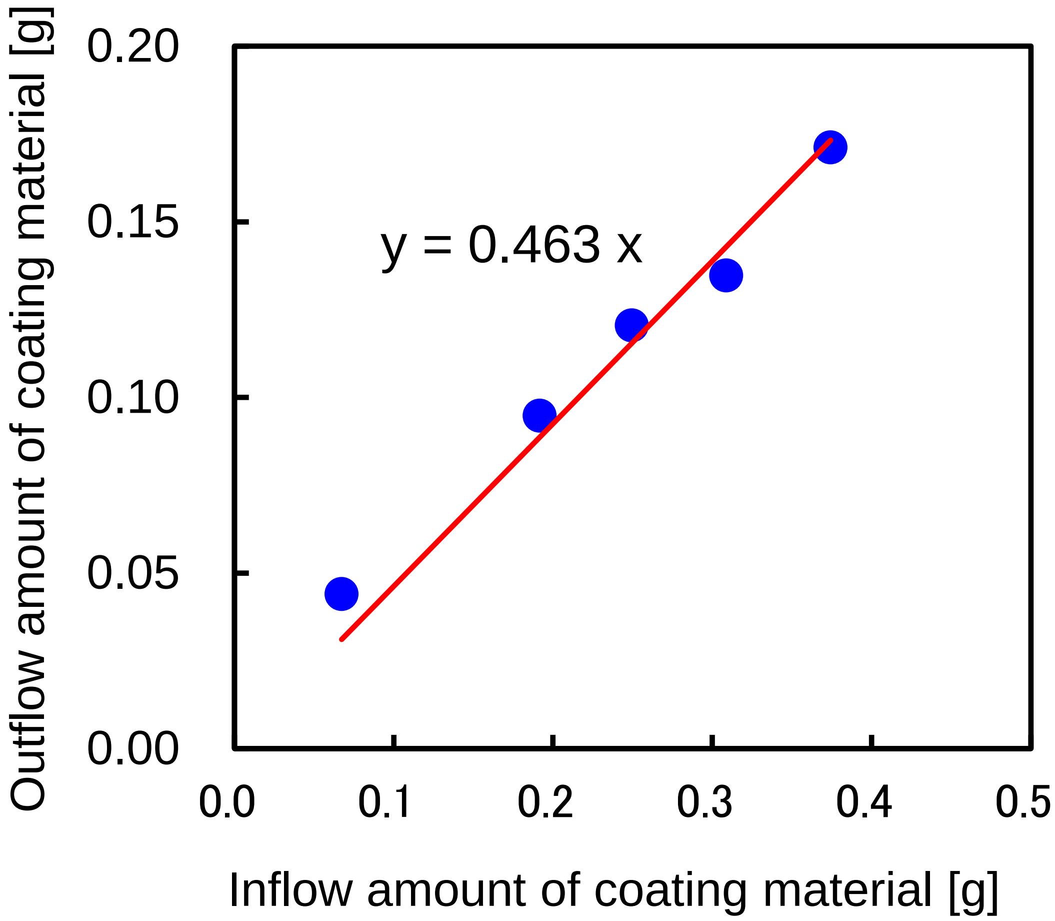

Figure S3. Relationship between the inflow and outflow amounts of coating material

Table S1. Solubilities of five silicone resins in supercritical CO_2

Sample N0.	Temp. [℃]	Pressure [MPa]	Solubility [g/L]		Temp. [℃]	Pressure [MPa]	Solubility [g/L]
S1	40	10.13	24.995		60	10.25	12.468
		12.08	27.515			12.19	15.090
		14.98	28.938			14.91	17.742
		19.87	30.935			19.46	20.779
		24.69	31.483			24.58	22.628
		29.68	31.743			29.52	24.165
S2	40	9.88	0.290		60	14.95	0.358
		11.76	0.466			20.09	0.716
		14.58	0.620			24.68	0.961
		19.33	0.778			29.77	1.196
		24.31	0.932				
		29.42	1.046		80	15.08	0.115
						19.73	0.379
						24.88	0.728
						29.03	0.973

Sample N0.	Temp. [℃]	Pressure [MPa]	Solubility [g/L]		Temp. [℃]	Pressure [MPa]	Solubility [g/L]
S3	60	14.92	0.101		80	19.79	0.142
		19.52	0.291			24.51	0.323
		24.62	0.452			30.07	0.535
		30.00	0.597				
S4	60	14.91	0.062		80	19.80	0.074
		19.62	0.152			24.43	0.167
		24.80	0.252			29.54	0.263
		29.98	0.343				
S5	60	19.72	0.030		80	19.95	0.024
		24.17	0.051			24.39	0.049
		29.43	0.080			29.54	0.094

Table S2. Numerical values used in the model to calculate the coating film thickness

Symbol	Item	Values
d_h	Diameter of ferrite particle	35 μm
d_v	Inner diameter of coating vessel	5.0 cm
l_h	Charged amount of ferrite particles	2.5 g
k	Proportional constant of outflow	0.463
L	Depth of coating vessel	6.5 cm
α	Chrastil's constant	0.6715
β	Chrastil's constant	1356
γ	Chrastil's constant	-5.437
ρ_c	Density of coating resin	1.42 g/cm^3
ρ_h	Density of ferrite particle	4.89 g/cm^3
